# Evaluation of the Hologic Panther Fusion investigational use only assays for gastrointestinal bacterial pathogens

**DOI:** 10.1128/jcm.00730-25

**Published:** 2025-11-14

**Authors:** Rama R. Yakubu, Jacob Merwede, Brianna Viglione, Vikas Patel, David R. Peaper

**Affiliations:** 1 Department of Laboratory Medicine, Yale University 5755, New Haven, Connecticut, USA; 2 Department of Laboratory Medicine, Yale New Haven Hospital 25047, New Haven, Connecticut, USA; Cleveland Clinic, Cleveland, Ohio, USA

**Keywords:** multiplex, molecular, diarrhea, enteric, stool pathogens

## Abstract

Historic detection methods for diarrheal pathogens are time-consuming, laborious, and may lack specificity and sensitivity. Multiplexed molecular panels for enteric pathogens are widely used for rapid and accurate results. We evaluated the performance of the investigational use only (IUO) Hologic Panther Fusion GI Bacterial and Expanded Bacterial assays (Fusion GI Bac), the Verigene Enteric Pathogens (EP), and BioFire FilmArray Gastrointestinal (BioFire GI) panels for the detection of primary bacterial causes of gastrointestinal infection. We conducted a multi-platform evaluation for the detection of *Salmonella*, *Shigella*/Enteroinvasive *Escherichia coli*, *Campylobacter*, Shiga toxin-producing *E. coli*, *Vibrio*, and *Yersinia enterocolitica*. Limited analysis of *Plesiomonas shigelloides* and *E. coli* O157 was also performed. A total of 591 stool specimens underwent complete analysis: 263 frozen positive specimens and 328 freshly collected samples. We used a 2-out-of-3 consensus as the reference for assay performance. Positive and negative percent agreement (PPA and NPA) were high for most pathogen/assay pairs. Individual false-positive (FP) or false-negative (FN) results were seen for all pathogens and assays. PPAs below 95% in the retrospective samples were seen for Shiga toxin (Verigene EP, 89.3%) and *Y. enterocolitica* (Verigene EP, 88.9% and Fusion GI Bac, 72.2%). There were 12 FP results for *Vibrio* affecting all assays. Factors contributing to erroneous results, including freeze/thaw effects, are discussed. Overall, the IUO Panther Fusion GI Bacterial and Expanded Bacterial assays were comparable to two commercially available GI panels with a straightforward workflow using a high-throughput instrument.

**IMPORTANCE:**

Bacterial causes of diarrhea lead to significant morbidity and mortality around the world. Historic testing methods, for example, antigens and culture. For these reasons, molecular testing for enteric bacterial pathogens has become widely used, but there are limited numbers of commercially available tests on the market, especially those suitable for higher-throughput testing. We show that the high-throughput, random- access investigational use only Hologic Panther Fusion Gastrointestinal (GI) Bacterial assay and Panther Fusion GI Expanded Bacterial assay perform comparably to existing assays.

## INTRODUCTION

Infectious diarrhea causes significant morbidity and mortality worldwide. Identifying the etiologic agent of diarrhea is important for the treatment of individual patients and public health monitoring and interventions. Historically, stool culture using specific selective and differential agars with subsequent morphologic, biochemical, and/or immunological analyses was the primary method of isolating and identifying enteric bacterial pathogens. However, these methods are time-consuming, laborious, costly, and may lack sensitivity (1). The specificity of selective culture is also low, necessitating subculture and subsequent workup to identify infrequently encountered pathogens, potentially delaying diagnosis and treatment.

Recent technical advances have led to the use of highly multiplexed FDA-cleared molecular assays capable of simultaneously detecting multiple pathogens in a single test. These panels have been designed to detect common etiologic bacterial, viral, parasitic, and fungal agents known to cause gastroenteritis, respiratory infections, bacteremia, or meningoencephalitis (2).

Clinical laboratories are increasingly using multiplexed molecular assays for the detection of gastrointestinal (GI) pathogens to overcome the limitations of culture-based methods. There are several commercially available tests for the detection of common enteric pathogens, including the Verigene Enteric Pathogens Test (Verigene EP), the BioFire FilmArray Gastrointestinal Panel (BioFire GI), Qiagen QIAstat-Dx, Luminex xTag Gastrointestinal Pathogen Panel (GPP), Biocode GPP, and BD Max Enteric Panels in the United States. These panels are associated with several key advantages over historical techniques: (i) significantly decreased turnaround time to results reporting, (ii) detection of coinfections with other pathogens assayed on the same panel, (iii) excellent negative predictive value, preventing unnecessary treatment and infection control precautions, (iv) generally high sensitivity and specificity, (v) more timely implementation or withholding of appropriate empiric antimicrobial therapy, and (vi) reduced hands-on time and associated labor costs for medical laboratory scientists (3, 4).

We evaluated the Verigene Enteric Pathogens Test (Verigene EP) (Diasorin), BioFire GI Panel (BioMerieux), and new investigational use only (IUO) Panther Fusion GI Bacterial assay and Panther Fusion GI Expanded Bacterial assay (Fusion GI Bac) (Hologic, Inc.) for the detection of the following diarrheagenic bacteria: *Campylobacter, Salmonella,* Shiga toxin-producing *Escherichia coli* (STEC)*, Shigella/Enteroinvasive E. coli (EIEC), Vibrio,* and *Yersinia enterocolitica*. All three panels are performed on different platforms and test for different combinations of bacterial, viral, or parasitic GI pathogens. All three assays are multiplexed PCR methods, but the approach used by each test system to detect multiple pathogens varies. All have significantly faster turnaround time than traditional stool culture, which can take up to three days for a negative result from some media.

The BioFire GI and Verigene EP panels were previously assessed in multicenter evaluations and in comparison with each other (5–7). In all cases, performances were generally comparable, with some variability observed in the sensitivity and specificity for some of the assayed targets. To our knowledge, this study is the first single-institution comparative study of the Verigene EP, BioFire GI, and Fusion GI Bac panels.

## MATERIALS AND METHODS

### Study design

We conducted a single-center, mixed retrospective/prospective, multi-platform evaluation of the bacterial targets on three GI panels: Panther Fusion GI Bacterial assay and Panther Fusion GI Expanded Bacterial assay (Fusion GI Bac) (Hologic, Inc., San Diego, CA), BioFire GI Panel (BioMerieux, Salt Lake City, UT), and Verigene Enteric Pathogens Test (Verigene EP) (Diasorin, Austin, TX) panels for the detection of *Campylobacter*, *Salmonella*, EIEC/*Shigella*, STEC, *Vibrio*, and *Y. enterocolitica*. We further evaluated the Fusion GI Bac expanded assay and BioFire GI for the detection of *E. coli* O157 and *Plesiomonas shigelloides*. No new specimens were collected solely for this study. Panel composition and method overview are shown in Table 1.

**TABLE 1:** Pathogen targets detected by each of the three panels evaluated in the study

		Hologic Panther Fusion GI bacterial & expanded bacterial assays	Verigene enteric pathogens	BioFire FilmArray GI panel
Detection Methodology		Multiplexed Real-Time PCR x2 Reactions	RT-PCR with bead nano array detection	Multiplexed RT-PCR with array detection and melt curve analysis
Primary targets included in the study*^a^*	Fusion GI Bacterial	*Campylobacter* spp: *C. coli, C. jejuni*	*Campylobacter* spp: *C. coli, C. jejuni, C. lari*	*Campylobacter* spp: *C. coli, C. jejuni, C. upsaliensis*
		*Salmonella* spp.	*Salmonella* spp.	*Salmonella* spp.
		Shiga toxin-producing *E. coli* (STEC)	Shiga toxin 1 (stx1) gene and/or Shiga toxin 2 (stx2) gene	Shiga toxin-producing *E. coli* (STEC) stx1/stx2
		Shigella/EIEC	*Shigella* spp.	Shigella/EIEC
	Fusion GI Expanded Bacterial	*Vibrio* spp: *V. parahaemolyticus*, *V. vulnificus*, *V. cholerae*	*Vibrio* spp: *V. parahaemolyticus*, *V. cholerae*	*Vibrio* spp: *V. parahaemolyticus*, *V. vulnificus*, *V. cholerae^b^*
		*Yersinia enterocolitica*	*Yersinia enterocolitica*	*Yersinia enterocolitica*
Secondary targets with descriptive data		*E. coli* O157	None	*E. coli* O157
		*Plesiomonas shigelloides*		*Plesiomonas shigelloides*
Pathogen targets not included in the current analysis		None	Norovirus Rotavirus	*Clostridiodes difficile* (toxin A/B) *E. coli* (EAEC) *E. coli* (EPEC) *E. coli* (ETEC) lt/st Parasites^*c*^ Viruses^*d*^

^*a*^ Samples with known species (by culture/sequencing) that were not common across all platforms were excluded from the study.

^*b*^ There is a specific *V. cholerae* call-out in BioFire GI.

^*c*^ Parasites included in BioFire GI Panel: *Giardia lamblia*, *Cryptosporidium*, *Cyclospora cayetanensis*, and *Entamoeba histolytica*.

^*d*^ Viruses included in BioFire GI Panel: Adenovirus F 40/41, Astrovirus, Norovirus GI/GII, Rotavirus A, Sapovirus.

### Inclusion and exclusion criteria

There were no restrictions on inclusion based on patient age, collection location, or inpatient or outpatient status. Specimens were not eligible for inclusion if they did not meet criteria for clinical testing, including leaking specimens, storage conditions exceeding acceptable specifications, unacceptable collection method, inadequate volume, formed stool, or specimens submitted on patients admitted >72 hours. Specimens that did not meet clinical criteria were not tracked further.

Additionally, initially eligible samples were excluded from final analysis if all testing could not be completed due to invalid results (*n* = 3) or lack of inclusion of an identified species in assay targets (*n* = 1, *Campylobacter lari*). Finally, retrospective samples testing initially positive on Verigene EP during standard of care (SOC) testing with culture-confirmed results that were negative for all pathogens by both BioFire GI and Fusion GI Bac were excluded (*n* = 5; *Campylobacter n* = 1, *Salmonella n* = 2, *Y. enterocolitica n* = 2) from further analysis.

### Specimen collection

Stool pathogen testing orders were at the discretion of the clinical care team. Stool specimens were collected according to standard stool collection and handling procedures. Specimens were submitted to the laboratory as either raw stool or in Cary-Blair media; raw stool specimens were transferred into Cary-Blair media according to the manufacturer’s instructions by the laboratory. Fecal Swab (Copan, Murrieta, CA) and traditional Cary-Blair media (Remel, Lenexa, KS) were used for the samples in this study.

### Study workflow

For the retrospective arm, a laboratory data extract identified samples testing positive by SOC Verigene EP for any enteric bacterial pathogen from 2019 to 2022. Frozen eligible samples were assessed for adequate volume, and results of SOC testing, including contemporaneous culture and public health laboratory testing, were recorded with a unique study identification number. An aliquot (1 mL) of Cary-Blair was labeled with the study identification number for BioFire GI and any required supplemental testing. An Aptima Multitest Swab (Hologic, Inc.) sample was prepared from the original Cary-Blair specimen and was labeled with the study identification number and prepared for Fusion GI Bac testing (see below). The original Cary-Blair sample was returned to the freezer.

For the prospective analysis, samples submitted to the laboratory for enteric pathogen testing as part of clinical care from mid-June 2023 to mid-July 2023 were eligible for inclusion as described above. Included specimens were an unselected convenience sample based upon clinical laboratory workflow testing demands and the ability to complete study testing within the time frames required by each manufacturer’s instructions for use. Upon receipt and preparation of the Cary-Blair sample as needed for clinical testing, a unique study number was assigned, and a Cary-Blair aliquot (1 mL) and Multitest sample were prepared with the study identification number as above. SOC testing, including any reflex cultures, was performed per standard procedure, and the results of SOC testing were recorded under the unique study number. The link between specimen identification and study identification number was broken. Subsequent testing for study purposes was performed using the de-identified Cary-Blair aliquot.

### Specimen testing

All initial testing on the Verigene EP was performed consistently with the manufacturer’s instructions as part of SOC testing. BioFire GI testing was performed on the de-identified Cary-Blair aliquot. All testing was performed within 96 hours of preparation of Cary-Blair, and testing was consistent with the manufacturer’s instructions except for preparation of Cary-Blair aliquots from frozen samples in the retrospective arm. For testing with Fusion GI Bac assays, a sample was prepared by entirely submerging the swab from an Aptima Multitest Collection Kit (Hologic, Inc.) into thoroughly mixed stool in Cary-Blair media, transferring the swab to the Multitest tube, swirling five times, breaking the swab at the score point, and re-capping with a pierceable cap (“Multitest samples”). Fusion GI Bac testing was performed on de-identified Multitest samples for the Bacterial and Expanded Bacterial assays on the Panther Fusion instrument simultaneously and according to the manufacturer’s instructions, except for preparation of Multitest samples from frozen samples in the retrospective arm. Multitest samples were prepared promptly upon thawing of frozen samples or preparation of Cary-Blair for fresh samples.

Per standard protocol at Yale New Haven Hospital, all specimens testing positive for any enteric bacterial pathogen by SOC test (Verigene EP) were reflexively cultured from fresh Cary-Blair specimens on selective and differential media appropriate for the recovery of the identified enteric pathogen(s) including Campy Agar, Hektoen Enteric agar, Xylose-Lysine-Deoxycholate agar, sorbitol-MacConkey agar, Thiosulfate-Citrate-Bile salts-Sucrose agar, Cefsulodin-Irgasan-Novobiocin Agar, or Gram-Negative Broth +STX-1/2 EIA for *Campylobacter*, *Salmonella*, EIEC/*Shigella*, STEC or *E. coli* O157, *Vibrio*, *Y. enterocolitica*, or STX-1/2. Culture was performed promptly after SOC molecular testing on fresh samples. During the prospective testing arm, samples generating a new positive result on either BioFire GI or Fusion GI Bac were set up for culture from de-identified, fresh Cary-Blair aliquots. Molecular retesting of some samples was performed to investigate discordant results, but the results of initial molecular testing were used for the calculation of statistics.

### Analysis and statistics

We used a 2-out-of-3 molecular consensus to define the reference result, and positive percent agreement (PPA), negative percent agreement (NPA), and 95% confidence intervals were calculated for each method in the retrospective and prospective arms. Fleiss’ Kappa statistics for agreement among all three methods were calculated, and 95% confidence intervals were calculated by bootstrapping with replacement with 1,000 iterations. Kappa statistics for the two methods were calculated when Fleiss’ Kappa was <94%.

## RESULTS

### Overall results for primary pathogens

**Fig 1:**
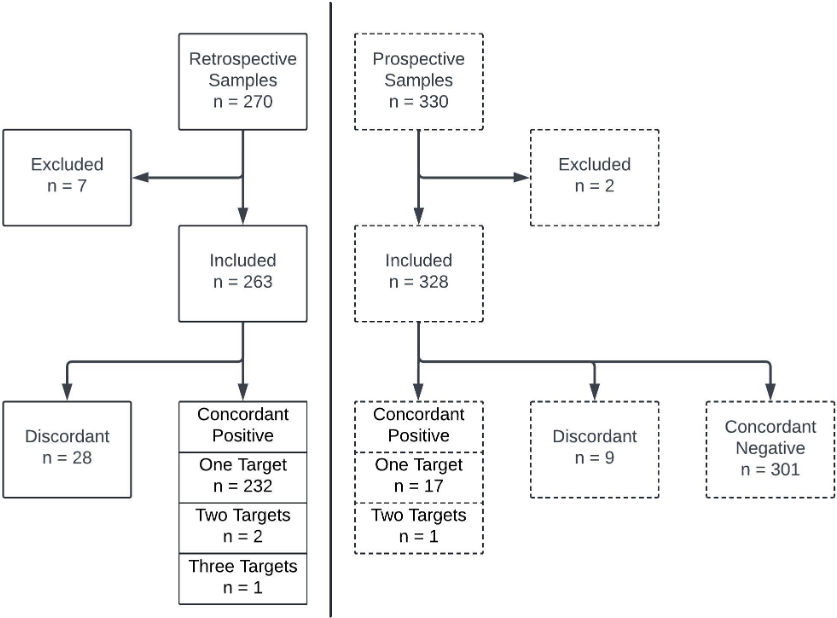
Overall results of testing in the retrospective (solid line) and prospective (dashed line) arms of the study. A total of 7 samples were excluded from the retrospective analysis for lack of species inclusion in all panels (*n* = 1), initial Verigene EP positivity with culture confirmation but negative results upon BioFire GI and Fusion GI Bac testing (*n* = 5), and invalid result/unable to resolve (*n* = 1). Two samples were excluded from the prospective arm for invalid results/unable to be resolved.

The overall results of testing for the retrospective and prospective arms are shown in Fig. 1. Testing was undertaken on 600 total specimens, with 7 and 2 specimens excluded from further analysis from the retrospective and prospective arms, respectively. Only samples testing positive during initial SOC testing were included in the retrospective arm, so there were no concordant negative samples. Twenty-eight samples were discordant, and 232, 2, and 1 were concordant by all three methods for one, two, and three pathogens, respectively. The triple-positive sample contained *Campylobacter*, EIEC/*Shigella*, and *Vibrio*, and the three double-positive samples each had *Campylobacter* in combination with EIEC/*Shigella*, Shiga toxin, or *Vibrio*. In the prospective arm, 301 samples were concordantly negative, with nine samples being discordant. Among concordant positive samples, 17 and 1 were concordant for one or two primary pathogens, respectively. The double-positive sample contained *Salmonella* and Shiga toxin.

### Retrospective analysis of primary targets

A total of 263 samples were included in the retrospective analysis, and 235 (89.3%) were concordant for all primary analytes tested. Using a 2-out-of-3 molecular consensus as the reference result, results of testing, including PPA and NPA, are shown (Table 2). *Campylobacter* and *Salmonella* were the most common pathogens detected, and *Vibrio* was the least frequently detected. PPA for pathogen/assay pairs was above 95% in all cases except for the following combinations: Shiga toxin/Verigene (89.3%), *Y. enterocolitica*/Fusion GI Bac (72.2%), and *Y. enterocolitica*/Verigene (88.9%). The NPA for all pathogen/assay pairs was above 99% in all cases except for *Vibrio* for both Fusion GI Bac (98.8%) and Verigene (97.7%).

**TABLE 2:** PPA and NPA for retrospective and prospective phases for shared primary bacterial enteric pathogens by method^*a*^^,^^*b*^

		Retrospective (*n* = 263)						Prospective (*n* = 328)						Overall (*n* = 591)
Pathogen	Method	Ref. Pos	Pos	PPA (%) 95% CI	Ref. Neg	Neg	NPA (%) 95% CI	Ref. Pos	Pos	PPA (%) 95% CI	Ref. Neg	Neg	NPA (%) 95% CI	Kappa 95% CI
*Campylobacter*	Fusion GI Bac	108	108	100 (96.6–100)	155	155	100 (97.6–100)	7	7	100 (64.6–100)	321	321	100 (98.8–100)	0.98 (0.96–0.99)
	BioFire GI		108	100 (96.6–100)		154	99.4 (96.4–99.9)		7	100 (64.6–100)		318	99.1 (97.3–99.7)	
	Verigene EP		107	99.1 (94.9–99.8)		155	100 (97.6–100)		6	85.7 (48.7–97.4)		321	100 (98.8–100)	
*Salmonella*	Fusion GI Bac	87	87	100 (95.8–100)	176	176	100 (97.9–100)	6	6	100 (61–100)	322	322	100 (98.8–100)	0.99 (0.98–1.0)
	BioFire GI		86	98.9 (93.8–99.8)		175	99.4 (96.9–99.9)		6	100 (61–100)		322	100 (98.8–100)	
	Verigene EP		87	100 (95.8–100)		176	100 (97.9–100)		6	100 (61–100)		322	100 (98.8–100)	
EIEC/*Shigella*	Fusion GI Bac	21	21	100 (84.5–100)	242	242	100 (98.4–100)	2	2	100 (34.2–100)	326	326	100 (98.8–100)	0.94 (0.87–0.98)
	BioFire GI		21	100 (84.5–100)		241	99.6 (97.7–99.9)		2	100 (34.2–100)		326	100 (98.8–100)	
	Verigene EP		20	95.2 (77.3–99.2)		242	100 (98.4–100)		2	100 (34.2–100)		326	100 (98.8–100)	
Shiga toxin	Fusion GI Bac	28	28	100 (87.9–100)	235	234	99.6 (97.6–99.9)	5	5	100 (56.6–100)	323	323	100 (98.8–100)	0.97 (0.92–1.0)
	BioFire GI		28	100 (87.9–100)		235	100 (98.4–100)		5	100 (56.6–100)		321	99.4 (97.8–99.8)	
	Verigene EP		25	89.3 (72.8–96.3)		235	100 (98.4–100)		5	100 (56.6–100)		323	100 (98.8–100)	
*Yersinia enterocolitica*	Fusion GI Bac	18	13	72.2 (49.1–87.5)	245	245	100 (98.5–100)	0	0	N/A^*c*^	328	328	100 (98.8–100)	0.81 (0.66–0.92)
	BioFire GI		18	100 (82.4–100)		245	100 (98.5–100)		0	N/A		328	100 (98.8–100)	
	Verigene EP		16	88.9 (67.2–96.9)		244	99.6 (97.7–99.9)		0	N/A		327	99.7 (98.3–99.9)	
*Vibrio*	Fusion GI Bac	6	6	100 (61–100)	257	254	98.8 (96.6–99.6)	0	0	N/A	328	327	99.7 (98.3–99.9)	0.59 (0.29–0.80)
	BioFire GI		6	100 (61–100)		256	99.6 (97.8–99.9)		0	N/A		327	99.7 (98.3–99.9)	
	Verigene EP		6	100 (61–100)		251	97.7 (95–98.9)		0	N/A		328	100 (98.8–100)	

^*a*^ For Verigene, reporting of either STX-1 or STX-2 was considered positive for Shiga toxin.

^*b*^ Kappa was calculated by the method of Fleiss for agreement among three or more independent evaluators for the entire data set of retrospective and prospective specimens. Confidence intervals around Kappa were calculated by bootstrapping.

^*c*^ N/A, not applicable.

### Prospective analysis of primary targets

Among the 328 specimens included in the prospective arm, 319 (97.3%) were concordant among all three methods, with 301 (91.8%) being concordantly negative. There were no samples considered reference positive for *Vibrio* or *Y. enterocolitica* by the 2-out-of-3 molecular assay consensus. There was a single false-negative (FN) result for *Campylobacter* by Verigene, giving a PPA of 85.7%; all other PPAs were 100% for all relevant pathogen/assay combinations. NPAs for all pathogen/assay combinations were above 99.7% (i.e., one false positive [FP]) except for *Campylobacter*/BioFire (99.1%) and Shiga toxin/BioFire (99.4%) (Table 2).

### Expanded bacterial targets

Both the BioFire GI panel and Panther Fusion GI Expanded Bacterial assay include specific reports for *E. coli* O157 and *P. shigelloides*, while the Verigene EP does not. Thus, we could only compare the results of BioFire and Fusion for these pathogens. In the prospective arm, no samples tested positive for *P. shigelloides* by either BioFire or Fusion. In the retrospective arm, four samples were positive by both methods, and one sample was positive by BioFire only. All five samples were included in the retrospective arm due to initial detection of other pathogens. The four samples testing positive for *P. shigelloides* by both BioFire and Fusion were originally positive for *Campylobacter* (*n* = 3) or *Salmonella* (*n* = 1). The single sample testing *P. shigelloides* positive by BioFire only was originally positive for both *Vibrio* (*Vibrio cholerae*) and *Campylobacter* by all methods, including culture. Of note, this sample also tested positive for EIEC/*Shigella* by BioFire only.

The results of *E. coli* O157 testing are presented in Table 3. There were three samples positive for *E. coli* O157 by both BioFire and Fusion: one was in the prospective arm, and two were culture-confirmed. The mean Fusion *E. coli* O157 Ct value for these three samples was 22.0. Six samples were Fusion-positive and BioFire-negative or BioFire-N/A (BioFire reports *E. coli* O157 as not applicable, N/A, on Shiga toxin-negative samples). Among these six, one was from the prospective arm. Two were culture confirmed for *E. coli* O157, but one of these was a Shiga toxin-negative sample. The mean Ct value for the culture-confirmed *E. coli* O157 was 19.7, but for the discordant, non-culture-confirmed samples, the mean Ct was 38.6.

**TABLE 3:** Results of BioFire and fusion *E. coli* O157 specific testing. 582 samples were negative for *E. coli* O157 by both methods

	Fusion GI Ex Bac Pos/BioFire GI Pos	Fusion GI Ex Bac Pos/BioFire GI Neg or N/A^*f*^	
Total	3	6	
Retrospective phase	2	5	
Other target(s)^*a*^	0	4	
Shiga toxin^*b*^	3	1	
*E. coli* O157 culture confirmed	2	2^*c*^	
Ct values (Avg)	22.0	19.7^*d*^	38.6^*e*^

^*a*^ Other includes *Campylobacter*, *Vibrio,* or *Yersinia*.

^*b*^ Shiga toxin consensus reference positive.

^*c*^ *E. coli* O157 was recovered from re-culture of 1 specimen that was STX-negative; *E. coli* O157 was detected by the CT DPH lab.

^*d*^ Culture confirmed; Shiga toxin consensus reference positive.

^*e*^ Culture not confirmed.

^*f*^ The BioFire GI panel reports the result for the O157 assay as “not applicable” (N/A) when the BioFire STEC stx1/2 assay result is negative. Of the six discordant samples, five were N/A for O157 by BioFire.

The single case of *V. cholerae* was detected as *Vibrio* species by all three assays, with a specific *V. cholerae* call-out by the BioFire GI, the only assay with a specific *V. cholerae* call-out. Reflex culture was positive for 3 + *V. cholerae* (data not shown).

### Overall assay agreement for primary pathogens

We calculated Fleiss’ Kappa statistics, a measure of inter-assay agreement for more than two methods, for all three comparator assays for all primary pathogens on the combined retrospective and prospective data sets (Table 2). Kappa was ≥ 0.94, signifying a high degree of agreement, for *Campylobacter*, *Salmonella*, EIEC/*Shigella*, and Shiga toxin. For *Vibrio*, Kappa was 0.59 among all three assays, with individual Kappa statistics below 0.66 for all pairwise comparisons. Likewise, Kappa for *Y. enterocolitica* was 0.81 among all three assays, with pairwise Kappa values ranging from 0.70 (Fusion and Verigene) to 0.89 (Verigene and BioFire).

### Discordant results for primary pathogens

**Fig 2:**
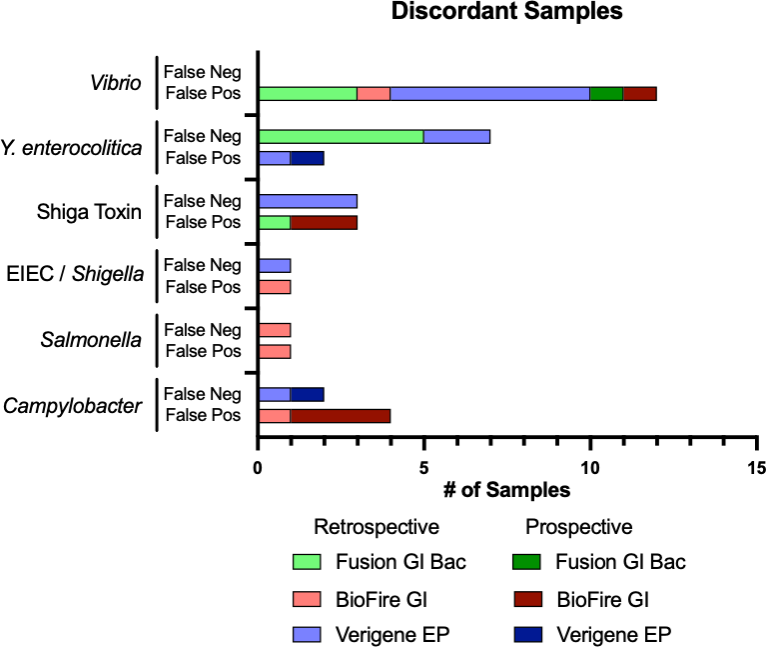
Discordant results by pathogen, platform, and study arm. A 2-out-of-3 molecular consensus was used to define a reference result for the determination of FP or FN results.

Results were considered discordant if they were not consistent with the 2-out-of-3 molecular assay consensus, resulting in 37 results being classified as either a FP or a FN result. There were 28 discordant results in the retrospective arm, with 15 FP results and 13 FN results (Fig. 2). Ten of the 15 FP results in the retrospective arm were seen for *Vibrio*, with all assays having FP results, with three, one, and six FP results for Fusion, BioFire, and Verigene, respectively. All three Fusion *Vibrio* FP samples were concordantly positive for *Campylobacter*, and the BioFire *Vibrio* FP sample was concordantly positive for EIEC/*Shigella. Vibrio* was not recovered from cultures of any of these specimens, and repeat Verigene testing from Cary-Blair aliquots during study performance was all negative (data not shown). A single FP result was seen for each of the remaining pathogens, with three from BioFire (*Campylobacter*, *Salmonella*, and EIEC/*Shigella*), and one each from Verigene (*Y. enterocolitica*) and Fusion (Shiga toxin). The BioFire and Fusion FP results were generated on specimens with concordant positive results of *Salmonella*, Shiga toxin, *Campylobacter*, and EIEC/*Shigella*, respectively.

Thirteen FN results were seen among retrospective samples, with 10 FN results coming from three pathogen/assay combinations: *Y. enterocolitica*/Fusion (*n* = 5), *Y. enterocolitica*/Verigene (*n* = 2), and Shiga toxin/Verigene (*n* = 3). Single FN results were seen for *Campylobacter*/Verigene, *Salmonella*/BioFire, and EIEC/*Shigella*/Verigene. Two of the five FN *Y. enterocolitica*/Fusion samples were positive for *Y. enterocolitica* by culture, and the single *Salmonella*/BioFire FN was culture positive for *Salmonella*. All other samples were culture-negative for their respective pathogens.

There were only nine discordant results among prospectively tested specimens, with 8 FP results and a single FN result (Fig. 2). The FN result was seen on the Verigene for *Campylobacter; Campylobacter* was not recovered in culture from this specimen. Among the 8 FP results, six were from BioFire with *Campylobacter* (*n* = 3), Shiga toxin (*n* = 2), and *Vibrio* (*n* = 1) being FP. There were single FP results for *Y. enterocolitica*/Verigene and *Vibrio*/Fusion as well. None of these organisms was recovered from culture.

### Multiple detections

There were 19 specimens, 18 of which were in the retrospective cohort, with at least one assay detecting multiple pathogens. Among these, seven were concordant positive for one pathogen with one assay detecting one additional pathogen. Seven samples were 3-out-of-3 concordant for one pathogen and 2-out-of-3 positive for a second pathogen. There was one sample with a 3-out-of-3 consensus for two pathogens and two additional detections by one assay. Three specimens were 3-out-of-3 concordant for two pathogens with no additional detections, and one specimen that was 3-out-of-3 concordant for three pathogens with no additional detections. All pathogens were detected in combination with other pathogens, but *Campylobacter* (*n* = 10), Shiga toxin (*n* = 8), and EIEC*/Shigella* (*n* = 7) were the most frequently involved pathogens (data not shown).

## DISCUSSION

A syndromic approach utilizing molecular panels for the diagnosis of gastrointestinal infections is becoming more widespread (8). Compared to traditional methods, multiplex molecular panels have a faster turnaround time, can more easily detect diverse pathogens that historically required multiple media or methods, can more readily detect polymicrobial infections, and demonstrate greater accuracy. Expedited results allow for earlier initiation of appropriate therapeutic agents targeting the specific pathogen detected or avoidance of inappropriate therapies for infections in which treatment is contraindicated (9).

The new Panther Fusion GI Bacterial and Panther Fusion GI Expanded Bacterial assays performed comparably to the two other commercially available molecular GI panels with a straightforward workflow using a high-throughput instrument. PPA and NPA for Fusion GI Bac assays were ≥98.8% for all analytes in prospective and retrospective arms, with the exception of *Y. enterocolitica* in the retrospective arm, for which PPA was 72.2%. The reduced PPA is likely multifactorial and may be due to freeze-thaw cycles of archived frozen samples compromising nucleic acid target stability, differences in amplification targets of the various assays, and/or other platform-dependent factors, as discussed below. Indeed, several frozen, retrospective samples with culture-confirmed results were excluded from the final analysis, as no pathogens were detected by the BioFire or Fusion assays.

The discordant results show a disproportionately high rate of FPs in both the retrospective and prospective arms. This is likely in part due to the 2-out-of-3 molecular assay consensus used for this study, which may inherently skew toward classifying results as FPs. This phenomenon is particularly pronounced in low-prevalence conditions such as infection with *Vibrio* or *Y. enterocolitica*. Other possible explanations include cross-reactivity of primers and probes with other targets, given that stool is a vastly complex matrix containing nucleic acids from other, potentially highly related, organisms; or some samples may have low-level target nucleic acids below the limits of detection (LODs) of the test and therefore are only detected sporadically. FNs are possibly due to the organism load in the study samples being close to or below the LOD of the test. There have been reports of potential cases of *Vibrio* false positivity associated with enteric panels in the past (10).

There were several discordant *E. coli* O157 results between the BioFire GI and Fusion GI Bac panels. As tested in this study, the Panther Fusion GI Expanded Bacterial assay did not incorporate the results of the Shiga toxin PCR from the Panther Fusion GI Bacterial assay into the reporting strategy, while the BioFire GI panel does, and reports the result for the O157 assay as “not applicable” when the STEC *stx*1/2 assay result is negative. If the Fusion assays incorporated a similar algorithm, this would have avoided four potentially FP results with high Ct values. One bona fide Shiga toxin-negative *E. coli* O157 would also have been missed, but the clinical significance of this organism is not clear (11, 12). The Fusion *E. coli* O157 assay is multiplexed with *Vibrio*, *P. shigelloides,* and *Y. enterocolitica* on the Panther Fusion GI Expanded Bacterial assay, and different approaches to result masking on the bacterial and expanded bacterial assays could be used on this platform if needed.

The effect of freeze/thaw cycles may have strongly affected the FN results for BioFire and Fusion in the retrospective arm of this study. We excluded five samples from analysis in the retrospective arm that were initially positive by Verigene during contemporaneous SOC testing and confirmation by culture. These samples all tested negative by both BioFire GI and Fusion GI Bac after thawing and preparation of new aliquots of Cary-Blair media. These included samples confirmed by culture to contain *Campylobacter* (*n* = 1), *Salmonella* (*n* = 2), and *Y. enterocolitica* (*n* = 2). Inclusion and classification of these samples as “True Positive” would not have substantially changed the calculated PPA and NPA values, with the exception of reducing PPA for BioFire and Fusion for *Y. enterocolitica* by 10% and 7.2%, respectively. However, these data clearly demonstrate the liability of nucleic acid targets in stool, especially after freezing, even when sufficient amounts of the organism were originally present to allow recovery by culture. Nucleic acid targets may be degraded as a result of frozen storage, contributing to FN results. Different assay design characteristics could lead to some assays being affected by these factors more than others. This highlights the inherent benefits and limitations of working with archived frozen specimens—while you can enrich for positive samples to more thoroughly interrogate positive agreement comparisons across platforms, it does not fully represent a real-world workflow in which samples are tested fresh, without frozen storage.

Gastrointestinal/enteric pathogen panels have been subject to a number of product notifications or recalls from the US Food and Drug Administration in recent years based on searches of the FDA Enforcement Report database. Recalls are addressing potentially FN or FP results across multiple platforms, including BioFire GI and Verigene EP, by themselves or in conjunction with specific types of Cary-Blair media or swabs (13). Some results in this study could have been affected by the issues raised in these notifications. Products that are more widely used are potentially more likely to generate concerns for erroneous results. Additionally, performance issues with highly multiplexed panels may be more likely to be identified due to the large number of analytes on a given panel. It is unclear whether the complexity of stool as a matrix, the relatedness of the stool microbiota and potential pathogens, the potential for DNA carryover from transport media manufacturing, or an interaction of these factors may affect these panels. In the right context, a positive result on an enteric pathogens PCR panel can help identify disease etiology, but results must be used in the context of a patient’s clinical signs and symptoms, epidemiological factors, and other lab results to help mitigate FN or FP results.

Factors to consider when implementing an enteric panel are: panel size and composition, ability to mask or suppress results, panel cost and reimbursement concerns, laboratory workflow, and potential access to Ct values. Both the Verigene EP and BioFire GI systems are amenable to rapid, on-demand/random-access testing in moderately complex laboratory settings. The Panther Fusion system requires a large instrument, but it has a flexible throughput with random access sample loading that allows for the processing of a larger number of samples with less hands-on time. The two Fusion GI Bac assays (Panther Fusion GI Bacterial assay and Panther Fusion GI Expanded Bacterial assay) may be run independently or simultaneously with each other or other assays on the same Panther Fusion instrument. Among the tested assays, only the Fusion GI Bac assays provide Ct values. Ct values can help investigate potential quality issues. The Verigene EP system supports result masking to tailor panels, and the Fusion GI Bacterial and Expanded Bacterial assays may be tested separately and also support result masking of specific targets to tailor the individual assays. Panther Fusion GI Viral and Panther Fusion GI Parasite assays are in development for a total of four 4–5 target panels for GI pathogens, as is the next generation of Verigene EP (Liaison Plex), but none of these are currently commercially available. Additionally, BioFire recently released a new panel with 11 targets. While panel cost is an important consideration for laboratories, much enteric pathogen testing is performed on outpatients, and rates of reimbursement may be more impactful to a positive business plan for implementation. In that regard, implementing a diagnostic stewardship approach with masking or cascading of results to meet certain clinical testing needs may be an important consideration for laboratories.

Empiric treatment for bacterial gastroenteritis is often not undertaken, but pathogen-specific therapy may be indicated in some cases (14). There were FP and FN results among all assays with all pathogens affected. Erroneous results could affect patient management, especially for patients with immunocompromise or severe disease, by (i) initiating incorrect empiric or targeted therapy, (ii) inappropriately extending or shortening diagnostic evaluations, (iii) incorrectly classifying patients subject to placement, residential, or work restrictions (e.g., foodservice workers), or (iv) inappropriately influencing public health reporting. However, clinical interpretation of multiplex molecular results must be tempered by clinical judgment. Detection of a pathogen does not establish causality, and negative results do not exclude infection when the pre-test probability remains high. Therefore, molecular findings should always be integrated with clinical presentation, epidemiologic context, and complementary clinical, laboratory, and/or ancillary data when making management decisions.

Our study has several limitations. We de-identified specimens in both arms of the study, and we could not link them back to patient records. This precluded the acquisition of more detailed clinical information, including duration of symptoms, risk factors, and treatment decisions that could have potentially informed some of the discordant results that we observed. Additionally, the use of a 2-out-of-3 molecular reference result is a potential source of bias. In the retrospective arm, samples were only included if they tested positive by Verigene through SOC testing, potentially positively favoring Verigene, and, in both arms, if one of the methods was substantially more analytically sensitive, it could appear as more FP results. Finally, while we tested archived known positive samples, the number of *Y. enterocolitica-* and *Vibrio*-positive samples included in the study was still low. Mis-categorization of one sample from these smaller groups would lead to greater changes in calculated test parameters as reflected in the wide confidence intervals for these analytes.

In conclusion, we describe the performance of the IUO Panther Fusion GI Bacterial and Panther Fusion GI Expanded Bacterial assays compared to the currently FDA-cleared BioFire GI and Verigene EP panels. There was a high rate of FN results from the Panther Fusion GI Expanded Bacterial assay for *Y. enterocolitica* when testing previously frozen samples, but the Fusion GI Bac PPA and NPA were high and comparable to the other assays for all other analytes in both prospective and retrospective testing. Choice of an enteric pathogen testing strategy involves consideration of many clinical, operational, and financial factors, and the Panther Fusion GI Bacterial and Expanded Bacterial assays support efficient random-access high-volume testing with flexible panel composition and performance comparable to currently available assays.
